# *N,N′*-Bis(3-methylphenyl)-*N,N′*-dyphenylbenzidine Based Distributed Feedback Lasers with Holographically Fabricated Polymeric Resonators

**DOI:** 10.3390/polym13213843

**Published:** 2021-11-06

**Authors:** Víctor Bonal, José A. Quintana, José M. Villalvilla, Pedro G. Boj, Rafael Muñoz-Mármol, Jose C. Mira-Martínez, María A. Díaz-García

**Affiliations:** 1 Departamento de Física Aplicada, Instituto Universitario de Materiales de Alicante, Universidad de Alicante, 03080 Alicante, Spain; victor.bonal@ua.es (V.B.); jmvs@ua.es (J.M.V.); rafa.marmol@ua.es (R.M.-M.); carlosmiramartinez@gmail.com (J.C.M.-M.); 2 Departamento de Óptica, Farmacología y Anatomía, Instituto Universitario de Materiales de Alicante, Universidad de Alicante, 03080 Alicante, Spain; ja.quintana@ua.es (J.A.Q.); p.boj@ua.es (P.G.B.)

**Keywords:** polymer films, organic distributed feedback laser, laser threshold, photostability

## Abstract

The molecule *N,N′*-bis(3-methylphenyl)-*N,N′*-dyphenylbenzidine (TPD) has been widely used in optoelectronic applications, mainly for its hole-transporting properties, but also for its capability to emit blue light and amplified spontaneous emission, which is important for the development of organic lasers. Here, we report deep-blue-emitting distributed feedback (DFB) lasers based on TPD dispersed in polystyrene (PS), as active media, and dichromated gelatin layers with holographically engraved relief gratings, as laser resonators. The effect of the device architecture (with the resonator located below or on top of the active layer) is investigated with a dye (TPD) that can be doped into PS at higher rates (up to 60 wt%), than with previously used dyes (<5 wt%). This has enabled changing the index contrast between film and resonator, which has an important effect on the laser performance. With regards to thresholds, both architectures behave similarly for TPD concentrations above 20 wt%, while for lower concentrations, top-layer resonator devices show lower values (around half). Remarkably, the operational durability of top-layer resonator devices is larger (in a factor of around 2), independently of the TPD concentration. This is a consequence of the protection offered by the resonator against dye photo-oxidation when the device is illuminated with pulsed UV light.

## 1. Introduction

Thin-film organic lasers (TFOLs) have received great attention in last decades as a result of their compactness and easy integration with other devices [1,2]. TFOLs can be pumped with low-power sources, in some cases with diode lasers or light emitting diodes, and very recently even with electricity [3]. Particularly, the distributed feedback (DFB) laser, consisting of a waveguide active film and a surface-relief grating as resonator, has been a successful TFOL mainly due to easy processability, chemical versatility, wavelength tunability and relatively low cost [4]. DFB lasers with one-dimensional (1D) gratings operating in the second-order of diffraction are attractive for certain applications, such as sensing [5,6,7], because the emission is perpendicular to the surface film and can be single mode.

**Figure 1:**
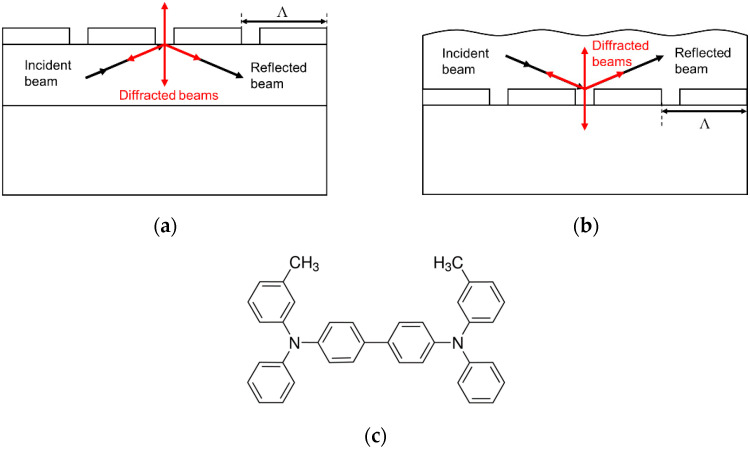
Scheme of a DFB laser with the DCG resonator located: (**a**) on top (RT), and (**b**) below (RB) the active film. The chemical structure of TPD is depicted in (**c**).

Many efforts in the field have focused on lowering the laser threshold (minimum pump energy required to operate), by either optimizing the active material [8] or the DFB resonator [9]. Lately, great attention is being devoted to all-solution processed devices, including both active film and laser resonator, pursuing truly low-cost and mechanically flexible systems [10,11]. A limitation of these all-solution processed lasers is that their performance is generally inferior to that of lasers based on high-quality gratings engraved on inorganic substrates. At this respect, DFB lasers based on dichromated gelatin (DCG) polymeric resonators have demonstrated great success with a wide variety of dyes emitting in different regions of the optical spectrum (visible, blue and deep blue, and near-infrared), particularly when the resonator is located above the active film (top-layer resonator architecture, denoted as RT, accounting for “resonator on top”, Figure 1a) [12,13,14,15,16]. This architecture is promising for the development of electrically pumped organic lasers because the grating spatially modulates the light emitted by the active material without the need of varying either the index or the thickness of the film, which remain constant. [17,18] On the other hand, devices with the DCG resonator located below the active film (below-layer resonator architecture, denoted as RB, accounting for “resonator below”, Figure 1b) showed significantly larger thresholds [12]. In this configuration, the active film surface is not planar, its thickness is difficult to control and it is not well-defined. The larger thresholds of the RB-based devices were explained by the low contrast between the refractive index of the resonator layer and that of the active film. Note that in all the DCG resonator-based lasers reported to date, the dye content was rather low (<5 wt%), so the refractive index of the active layer was approximately that of pristine polystyrene (PS).

In relation to the active materials, many compounds have been investigated [1,2,8]. In this context, the molecule *N,N′*-bis(3-methylphenyl)-*N,N′*-dyphenylbenzidine (TPD) (Figure 1c), which has been extensively used as a hole-transport material in the fabrication of organic light-emitting diodes (OLEDs) [19] and solar cells [20], has also received attention for its light emission properties [8,21]. The potential of TPD for laser applications was demonstrated in 2002 through the observation of amplified spontaneous emission (ASE) in PS films doped with TPD [22], followed by additional studies towards the optimization of various aspects [23,24]. An important characteristic of TPD is that it can be doped into the matrix at very high concentrations with practically no photoluminescence (PL) quenching. In fact, ASE was reported for TPD concentrations (in PS) between 2.5 and 100 wt% [24]. With regards to TPD-based DFB lasers, there are few reports in the literature, partly because of the rather poor photostability of TPD and its high sensitivity to air [8,25,26]. One of these devices, based on a resonator engraved on glass (and therefore, not all-solution processed) was developed in our laboratory. Even though the resonator profile of the devices was rather noisy, laser thresholds of 6 μJ/pulse and linewidths lower than 2 nm were measured.

Here, we report DFB lasers based on DCG resonators in both configurations (RT and RB), and using as active material TPD dispersed in PS at various concentrations (from 3 to 60 wt%). The fact that TPD can be doped at high concentrations in the matrix allows fabricating DCG-based DFB lasers with different properties than the ones previously reported. Particularly, this enables increasing the refractive index of the active layer and therefore the light confinement in the waveguide, which is a key to improving the performance of lasers with the RB configuration. Finally, the fact that the photostability of blue-emitting dyes, such as TPD, is inferior to that of dyes emitting at longer wavelengths, makes TPD especially suitable for comparing the possible differences between the two resonator configurations in adverse conditions.

## 2. Experimental Techniques for DFB Laser Fabrication

### 2.1. DCG Surface Relief Gratings Fabrication

Surface relief gratings for DFB laser resonators can be fabricated by various methods such as electron beam lithography (EBL), nanoimprint lithography (NIL) or holographic lithography (HL) [2,27]. EBL, which is considered to have the highest resolution and has the advantage of allowing many designs, but is quite expensive. NIL, which has received great attention in the last years for its potential in volume production, requires the use of a stamp master. In this case, gratings are regularly fabricated by thermal-NIL on a resist and then transferred to the substrate by ion-beam etching. The direct transfer onto the active films is also possible. Our group has reported efficient DFB lasers based on perylene derivatives with gratings imprinted by thermal-NIL [28]. The use of HL allows to easily modify the period and to fabricate gratings of larger dimensions (up to a few squared centimeters) than those prepared by EBL or NIL (generally around 2 mm^2^). Even though the quality is generally inferior to that obtained with EBL, we have developed a high resolution process to fabricate DCG gratings of the period required to obtain DFB lasing, typically between 500 and 200 nm (2000 and 5000 lines/mm) with relatively high quality [14]. The preparation and processing procedures used in this work to obtain surface-relief DCG gratings are indicated in detail in Table 1.

The thickness of the DCG film, which determines the grating depth, *d*, depends on gelatin concentration. Here we have used a concentration 2.2 wt% to obtain DCG films with a thickness of about 100 nm, so the final *d* value is around 100 nm.

**Figure 2:**
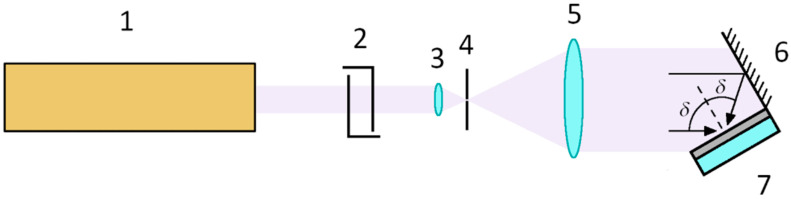
Setup for holographic recording of HL gratings in DCG: 1, argon-ion laser; 2, shutter; 3, microscope objective (40×); 4, pinhole (10 μm); 5, collimator (20 cm focal length); 6, mirror; 7, DCG film coated on a fused silica substrate (2.5 × 2.5 cm^2^). The angle between the mirror and the DCG plate is maintained constant and equal to 90°.

The experimental setup used regularly in our laboratory for exposing HL gratings is the wavefront-splitting Lloyd’s interferometer [29]. In this interferometer a mirror is placed perpendicular to the recording plate to produce a second beam (see Figure 2). The main advantage of this simple arrangement is that the grating period is easily changed by rotating the mirror-DCG plate assembly according to the equation:

Λ = *λ*/(2·sin*δ*), (1)

where *δ* is the angle between the two beams that interfere in the DCG plate. The optical system is very compact because it consists of only one mirror mounted rigidly attached to the DCG plate. However, the arrangement was set on a thermal and vibration isolation table to guarantee the stability of the system. When the light source is a laser with low coherence, fringe visibility decreases along the recording plate. This situation is relatively common when using high power or UV lasers. The case of reduced spatial coherence is particularly critical because of the superposition of different portions of the wavefront in the setup of Figure 2. So we reduced the diaphragm in the cavity of the Ar laser until only a single mode oscillates in the cavity. Concerning the temporal coherence, the fringes contrast is maximum in points of the DCG plate close to the mirror and decreases as it moves laterally. This is because the path traveled by the rays reflected in the mirror is longer than that of rays directly reaching the plate, and the path difference increases laterally from the edge of the plate close to the mirror (see Figure 2). In this work we have inserted a Fabry-Perot etalon in the cavity of the Ar laser for high temporal coherence. In addition, an absorbent plate is attached to the back side of the substrate with an index matching liquid to avoid lack of uniformity due to backward reflections.

Note that the desensitization time, 6 s, is relatively short. This time was found to be the minimum needed to remove the spectrophotometric signal corresponding to ammonium dichromate while maintaining resolution and layer uniformity. The most characteristic step of this process is the dry-development in an oxygen plasma because it allows for the obtaining of surface relief gratings with a period lower than 200 nm [14]. This aspect is advantageous in comparison to the wet processes used in conventional holography to generate volume patterns, which does not allow the recording of relief gratings with a period lower than 5 µm (200 lines/mm) [30]. Development was controlled by following the evolution of the diffracted blue light from a small diode laser. The signal increases until it reaches a maximum, which means that *d* approximately equals the DCG film thickness. Here, the thickness is 100 nm and the development time is between 8 and 9 min. Finally, it should also be noted that after the dry-development step, gratings are not only insoluble in most organic solvents (e.g., toluene), but also in water, so that they can be cleaned and reused.

**Figure 3:**
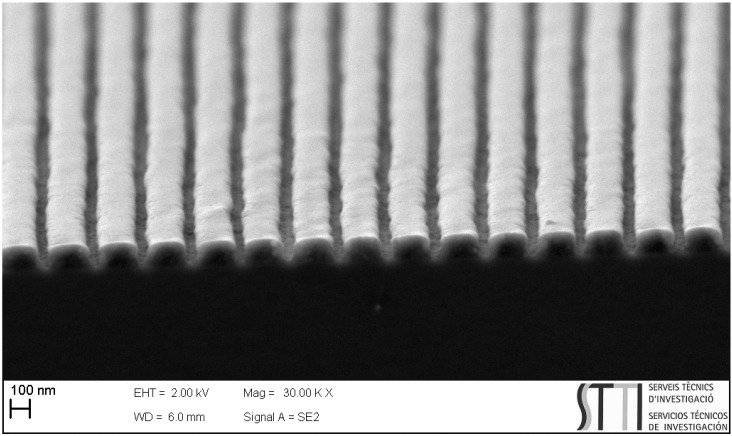
FESEM image of a dry developed DCG grating with Λ = 250 nm.

Figure 3 is a SEM image of a DCG grating with Λ = 250 nm fabricated with the described method. These gratings have a near-rectangular profile with a duty-cycle, defined as the ratio between the grating ridge width and the period, of approximately 0.75. In addition to the period, another important parameter is *d*. The DCG film thickness and consequently the *d* value, approximately 100 nm, obtained following the indicated procedure, can be modified by changing the gelatin concentration.

The diffraction efficiency of surface-relief gratings depends on the product of *d* and the refractive index difference, Δ*n*, between the index of the grating material and that of the medium in which it is immersed. In calculations, it must be taken into account that the refractive index of the dry-developed DCG is appreciably higher than that of the starting gelatin material and very similar to that of PS, as can be seen by the drastic decrease in diffracted light (about a factor of 100) when the grating is covered by a layer of this polymeric material.

### 2.2. Active Film Fabrication

The procedure to make the active film, PS doped with TPD, is indicated in detail in Table 2. Both reagents were obtained from Merck and used as received (PS average Mw 35,000 g/mol). Film transparency and homogeneity were good for concentrations of TPD up to 60 wt%. Films with higher concentrations of TPD presented inhomogeneity problems and neat films remained transparent just for a few hours after preparation. The percentage of PS in the solvent was adjusted to ensure that the active films support only the fundamental transversal electric and magnetic modes, TE_0_ and TM_0_, respectively. The extraction of the solvent was found to be a very important step to get low threshold and good stability of the DFB laser. The film’s refractive index and thickness were obtained from the transmission spectrum in the transparent spectral window by a method recently developed in our laboratory [31].

### 2.3. DFB Laser Assemmbling

In these DFB lasers, the light diffracted by the grating in the second order provides the feedback in the waveguide active layer, and that diffracted in the first order is the laser light emitted in a direction perpendicular to the film surface (see Figure 1a,b). The grating period was calculated by considering that the laser wavelength approximately satisfies the Bragg condition.

*λ*_Bragg_ = *n*_eff_ Λ, (2)

where *n*_eff_ is the effective refractive index of the waveguide that can be calculated by optical modeling, and Λ is the grating period.

Two laser architectures were fabricated in this work: (i) the top configuration, RT, with the grating located on top of the active film (see Figure 1a); and (ii) the below configuration, RB, with the grating located below the active film (see Figure 1b). We remember here that the implementation of the RT is possible because DCG is soluble in water and insoluble in organic solvents (used in most cases to prepare the active films). Thus, resist deposition does not damage the active layer. Another water-soluble resist material that has demonstrated success to fabricate RB-type organic DFB lasers is dichromated poly(vinyl alcohol) (DCPVA) [32]. Work towards the preparation of DFB lasers based on DCPVA resonators with TP configuration is currently under way.

### 2.4. Optical Characterization

Absorption spectra of active films were obtained using a Jasco V-650 spectrophotometer and PL measurements were made with a Jasco FP-6500 spectrofluorometer.

PL quantum yield (PLQY) measurements were made in active layers of thickness ~300 nm for the different TPD concentrations using a Jasco ISF-834 integrating sphere attached to the Jasco FP-6500 spectrofluorometer.

ASE characterization was performed using a Nd:YAG pulsed laser (10 ns, 10 Hz) operating at a UV wavelength of 355 nm, which is near the maximum absorbance of TPD. The energy of the pulses was varied using neutral density filters. Samples were pumped at normal incidence with a stripe beam (3.5 × 0.5 mm^2^) formed by a cylindrical lens, and the output light was collected from the edge of the film with an optical fiber coupled to an Ocean Optics USB2000 + UV–VIS fiber spectrometer with a spectral resolution of 0.6 nm.

DFB measurements were made by pumping at 355 nm with the same Nd:YAG pulsed laser used in the ASE measurements. The pump beam for characterizing lasers was polarized parallel to the grating lines and incident over the sample at a 30° angle with respect to the perpendicular to the film plane. Thus, the shape of the illuminated area on the sample is elliptical, with a minor axis of 1.0 mm. The emitted light was collected perpendicularly to the surface with the fiber spectrophotometer placed at about 1 cm from the sample.

## 3. Results and Discussion

### 3.1. Active Film Optical Properties

**Figure 4:**
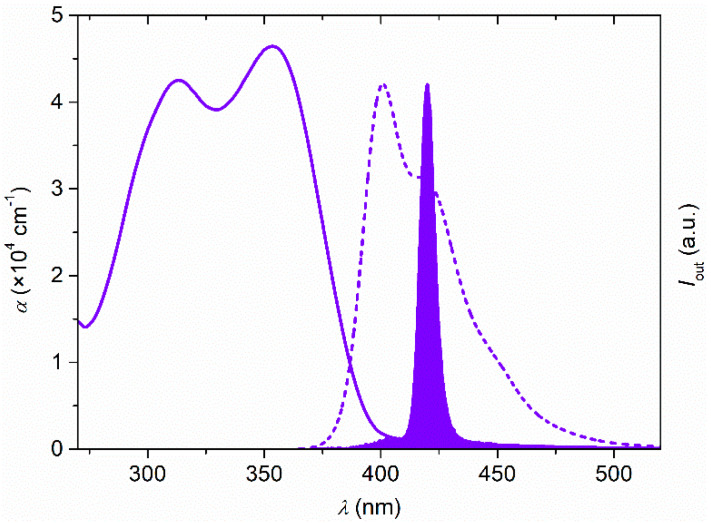
Absorption coefficient, α, PL intensity and ASE spectra of a 30 wt% TPD doped PS film: Absorption coefficient (solid line, left axis), PL (dashed line, right axis), and ASE, (filled area, right axis).

First, we consider the optical response of the active layer. Figure 4 shows the absorption and emission (PL and ASE) spectra of a 30 wt% TPD-doped PS film. ASE appears at around 420 nm with a linewidth of 6 nm as the pump energy increases over 50 µJ/cm^2^. A detailed discussion of the ASE properties of TPD-doped PS films and their dependence with the TPD concentration in the film was carried out by Calzado et al. [24].

### 3.2. Laser Threshold

DFB lasers based on PS doped with different concentrations of TPD (3, 7, 15, 30 and 60 wt%) were assembled in the two resonator RB and RT architectures. Gratings with Λ~270 nm were fabricated from DCG films of thickness of about 100 nm. Each device was repeated four times, with gratings of slightly different periods to obtain the laser emission as close as possible of the ASE peak, and thus to enable a better comparison of the responses of both configurations.

**Figure 5:**
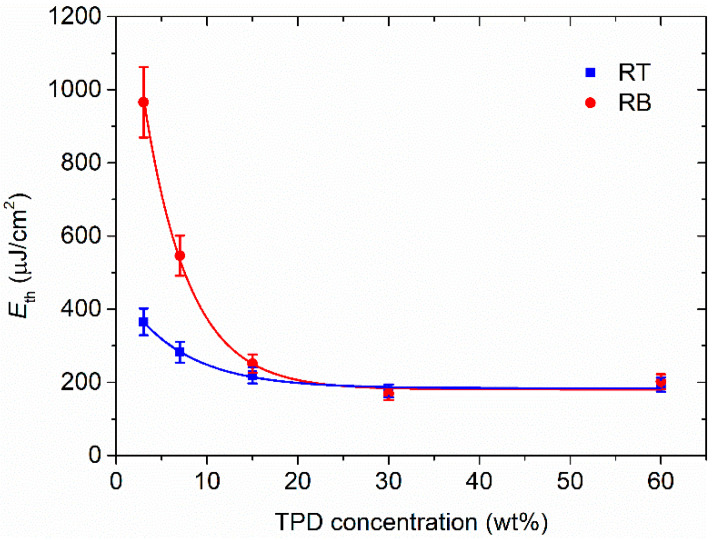
Thresholds of DFB lasers based on TPD for RB and RT configurations.

**Figure 6:**
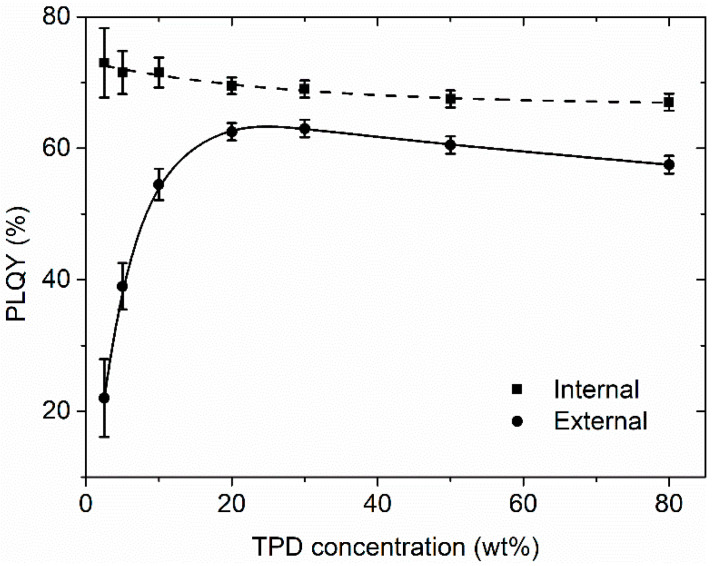
External PLQY (ratio between emitted and incident light) and internal PLQY (ratio between emitted and absorbed light) of active films of PS doped with different concentrations of TPD.

One of the most important parameters to assess the performance of a DFB laser is the threshold, i.e., the minimum pump energy density required to operate, *E*_th_. Threshold variations as a function of the TPD concentration for all those lasers are shown in Figure 5. It can be seen that the threshold decreases when the TPD concentration increases up to 20 wt%. Then it keeps a constant value of about 180 µJ/cm^2^. This behavior is similar to that of the ASE threshold found previously [24]. It is related to the efficiency of the PL process, when this is quantified through the external PLQY (i.e., ratio between emitted and incident light), see Figure 6: in the absence of PL quenching mechanisms, this parameter would increase with concentration simply because the number of emitters increases (absorption increases). The internal PLQY was also calculated taking into account absorbed light and included in Figure 6. On the other hand, the saturation of the threshold (and the external PLQY) above 20 wt% is due to molecular interaction. However, the type of interaction that takes place and why the PL is not completely quenched at high TPD concentrations as happens in most molecular laser dyes remains unclear.

**Figure 7:**
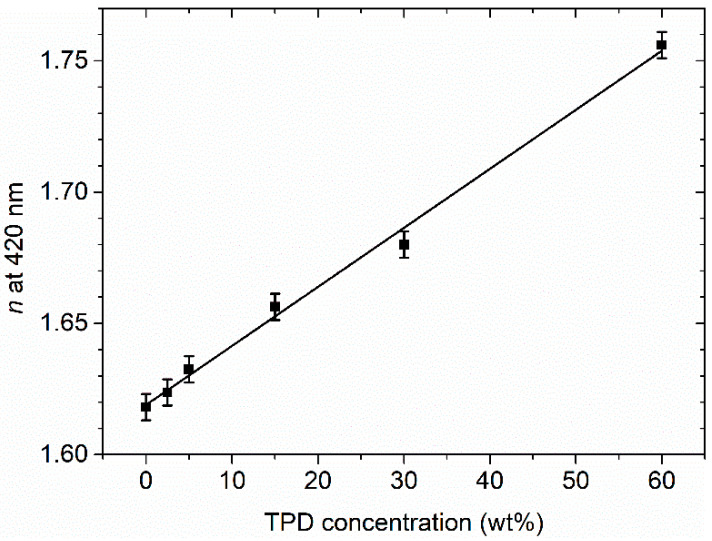
Refractive index of active films of PS doped with different concentrations of TPD.

In addition, Figure 5 shows that the threshold is appreciably higher in the RB configuration for concentrations lower than 20 wt%. In order to explain this difference, we consider that for low TPD concentrations, the index of the active film decreases and gets close to the value of undoped PS (see Figure 7), which in turn is similar to that of the developed DCG grating. Thus, in the RB configuration and for low TPD concentrations, the spatial index modulation becomes very small as a consequence of the decrease of *n* for the active film, becoming negligible for very low concentrations. So the effect of the grating in these cases is dominated by the apparently less important gain modulation mechanism [33]. On the other hand, in the RT configuration and for low TPD concentrations, the decrease of the active film index allows a larger amount of the emitted light to interact with the grating, whose effect is fairly large because it is immersed in air. When the TPD concentration increases, the index modulation increases, and thus the threshold becomes similar for both configurations.

**Figure 8:**
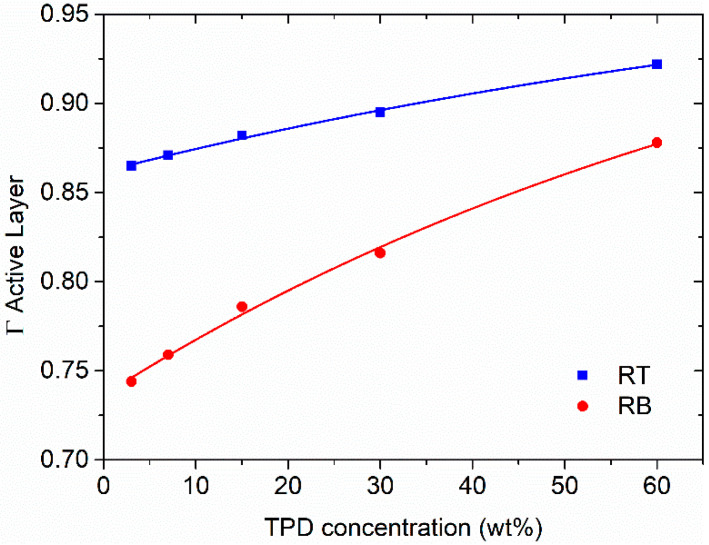
Optical electric field confinement factor, Γ, of the fundamental waveguide mode TE_0_ in the active film as a function of the TPD concentration for RB and RT configurations.

It is known that the lowest threshold is obtained when the fundamental TE_0_ mode is highly confined in the waveguide [34,35,36]. Thus, a parameter that can explain the threshold difference between the two configurations is the degree of confinement of the optical electric field in the active layer. Waveguide field confinement factor values, Γ, calculated through a 1-D multilayer slab waveguide mode solver program [37] are shown in Figure 8 and Figure S1 in the Supplementary Materials. Here, as in the case of external PLQY, a clear correlation between waveguide confinement and external PLQY is found, which is consistent with the fact that the higher the confinement the lower the threshold.

**Figure 9:**
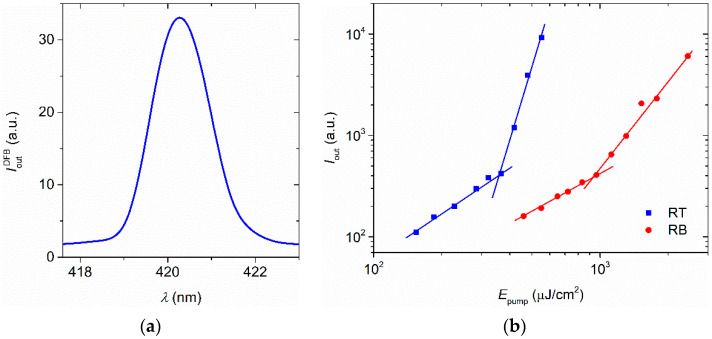
(**a**) Laser spectrum of a DFB device based on TPD (3 wt%). (**b**) Output intensity, *I*_out_, as a function of pump energy density, *E*_pump_, for two DFBs lasers based on TPD (3 wt%) with different configuration: RT (blue) and RB (red). Threshold energy densities were calculated from this graph as the energy density at which a drastic change in the slope curve occurs.

The DFB laser spectrum, which has a similar shape for all the prepared devices, is shown in Figure 9a. It is seen that emission is close to 420 nm. The reported linewidth, 1.3 nm, is limited by our USB spectrophotometer. The output intensity as a function of the pump energy density for DFBs lasers in both configurations are shown in Figure 9b for a TPD concentration of 3 wt%, for which the largest threshold differences between both configurations were observed. The threshold energy density, *E*_th_, was calculated from these graphs as the energy density at which a drastic change in the slope curve occurs [38].

It is interesting to know whether the different behavior shown by the RT and RB configurations at low dye concentrations is applicable to other laser dyes different than TPD. Generally, the amount of dye that can be introduced into the polymer matrix to conform the active layer is very small (typically below 5 wt%), due to PL quenching. Here, we compare the results obtained with DFB lasers in both configurations using the commercial perylene derivative known as perylene orange, (PDI-O). Perylene dyes have been widely used as active materials in the fabrication of TFOLs due to their low threshold and high photostability [38,39,40]. In the prepared devices the active layer consists of PS doped with 1% of PDI-O. DCG gratings were fabricated to have a period of 372 nm so they diffract light of 580 nm and have a depth of 100 nm. The active films were prepared to have a thickness of 600 nm, so they support only the TE_0_ mode. The fabrication of two devices, with RB and RT configuration, was carried out by following the same techniques than those described in this paper to prepare the TPD lasers. Remarkably, we found that the threshold in the RT configuration, 15 µJ/cm^2^, is about three times lower than that in the RB one, 40 µJ/cm^2^, in accordance with results obtained when working with TPD.

### 3.3. Photostability

**Figure 10:**
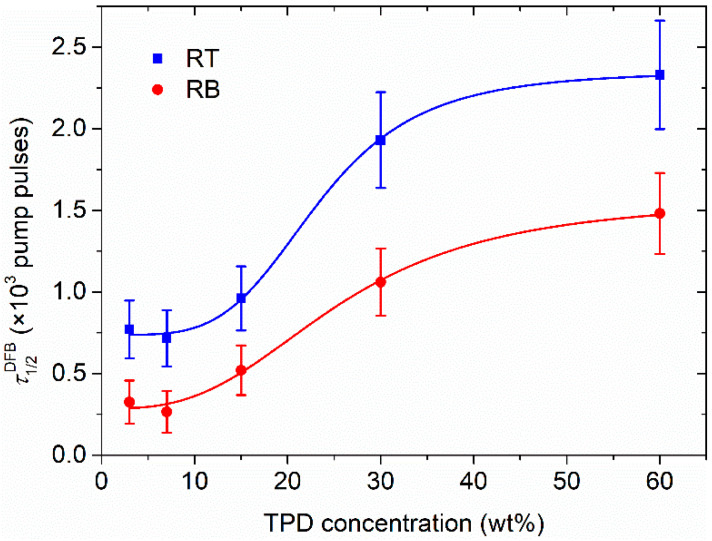
Photostability of DFB lasers based on TPD for RB and RT configurations.

The photostability of the prepared lasers was also studied. The DFB operational lifetime can be characterized by the photostability half-life, *τ*_1/2_, defined as the time (or the number of pump pulses) at which the emitted DFB intensity decays to half of its initial value. Figure 10 shows measurements made in ambient air conditions under a UV pump energy of ~15 mJ/cm^2^ at 355 nm (more details in Figure S2 in Supplementary Materials). It can be seen that *τ*_1/2_ increases with concentration as it happens with the ASE intensity when an active film is irradiated [24]. Therefore, the increase of photostability is attributed to the fact that the number of active molecules is bigger. The value of *τ*_1/2_ corresponding to a laser in RT configuration based on a 30 wt% TPD-doped film is approximately 2 × 10^3^ pump pulses (3.3 min). By comparing results obtained in the two configurations, a better photostability is found in the RT top one by a factor of almost two.

**Figure 11:**
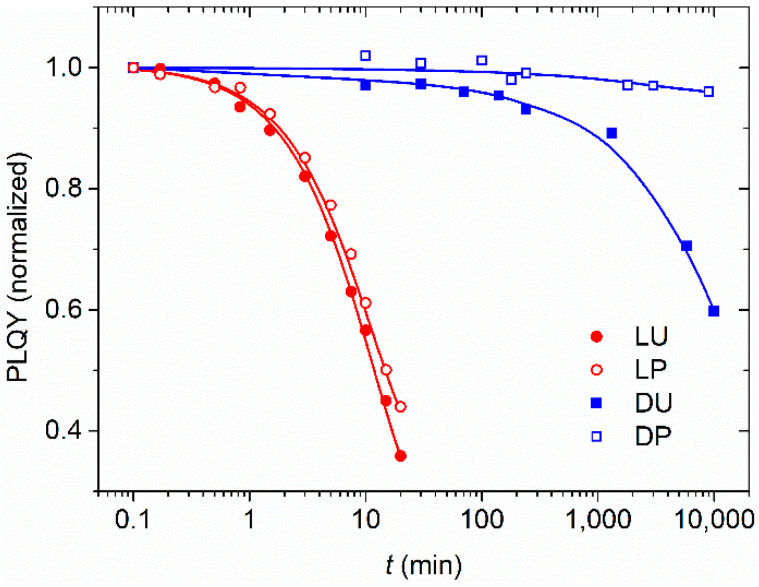
Normalized PLQY of an unprotected 30 wt% TPD doped PS active film stored in the dark (DU, blue full squares) or irradiated under UV pulsed light at 355 nm (LU, red solid circles). Experiments were repeated in samples with a protecting layer of dry-developed DCG in the dark (DP, blue empty squares) and irradiated with UV pulsed light (LP, red empty circles).

In order to relate these results to the speed degradation of TPD, we have compared the decrease of the internal PLQY of unprotected 30 wt% TPD doped PS active films under two circumstances: kept under dark storage; and subjected to a 95 mJ/cm^2^ pulsed UV illumination at 355. Besides, measurements were repeated by depositing a 100 nm layer of uniformly exposed and developed DCG on top of the TPD-doped PS films. We found that the initial value of the internal PLQY for unprotected films was approximately 67%. Figure 11 shows the time evolution of films in the different conditions. Values were normalized to compare protected with unprotected films. Samples were observed to keep well at room temperature (23 °C) in the dark for at least one month when they are protected, while the storage time without observing changes is reduced to one week when they are unprotected. Concerning the stability of the TPD films, even though most studies have been done with neat films, crystallization processes have been recognized as the main source of degradation [41,42]. In addition, there is evidence that photo-oxidation processes, which take place upon film irradiation with UV light in air, produce impurities that cause the PL quenching of TPD [43]. In fact, our results show that degradation is very fast under UV pulsed light. The PLQY value is reduced by half in about 10 min and even though protected films degrade more slowly, the factor seems to be smaller than two.

Finally, the photostability of lasers based on PDI-O was also studied. Since perylenes have very high *τ*_1/2_ values_2_, the duration of the experiments was shortened by pumping the samples with an extremely intense light, *I*_pump_ = 15 mJ/cm^2^, 1000 times more intense than its threshold. In these extreme pumping conditions, the value of *τ*_1/2_ corresponding to a RT laser was 1.5 × 10^4^ pump pulses, 3.8 times longer than that of the RB laser, 4 × 10^3^ pump pulses, in accordance with the results obtained with TPD-based lasers.

## 4. Conclusions

We have shown that in deep-blue surface-emitting DFB lasers based on TPD, the RT configuration has some advantages compared to the RB one. RT-type devices have been successfully assembled because of the immiscibility of the DCG and the polymer matrix of the active layer. The laser threshold decreases when the TPD concentration increases up to 20 wt%, and then keeps at a constant value of about 180 µJ/cm^2^. Besides, when concentrations are lower than 20 wt%, laser thresholds of RT-type devices are lower than those of the RB-ones. This effect seems to be due to a better field confinement in the active layer when the laser is in the RT configuration. The ratio between both thresholds increases as the TPD concentration in the film decreases, reaching a value of approximately two for a concentration of 5 wt%. The RT configuration also leads to a better photostability in a factor around two, independently of the TPD concentration. In this case, the effect is due to the protection offered by the DCG grating against dye photo-oxidation when the device is illuminated with pulsed UV light. Additionally, the validity of the method for dyes which can only be doped at low concentrations was verified by fabricating yellow emitting DFB lasers in the two configurations using perylene orange instead of TPD.

## Figures and Tables

**Table 1:** Procedures to fabricate relief gratings on DCG layers.

A. DCG film preparation
(1) Swell inert gelatin (Rousselot 200 bloom) in distilled water at room temperature (23 °C) for 2 h. (2) Dissolve gelatin under mild agitation; Increase temperature at an approximate rate of 1 °C/min to 60 °C and then decrease temperature at same rate to 50 °C. (3) Sensitize with ammonium dichromate (Merck), 35 wt% with respect to gelatin. (4) Deposit the sensitized solution at 40 °C on a FS substrate (2.5 × 2.5 cm^2^) or an active layer by spin-coating (3000 rpm in a LabSpin6 SÜSS Microtec machine).
B. Exposure
(1) Expose to saturation (average exposure of 45 mJ/cm^2^) in the Lloyd’s configuration with light of a wavelength of 364 nm provided by an Ar laser (Coherent Innova 308C Argon Ion Laser) polarized perpendicular to the incidence plane.
C. Desensitization
(1) Wash samples with mild agitation in a cold water bath (15 °C) of distilled water for 6 s. (2) Centrifuge the sample at 500 rpm for rapid drying.
D. Development
(1) Dry-develop in an oxygen plasma using a surface treatment machine (Diener Zepto). Development is controlled by measuring the time evolution of the diffracted intensity.

**Table 2:** Procedures for preparing the active film.

(1) Dissolve PS and TPD in toluene solution under mild agitation overnight. The concentration of TPD is varied between 3 and 60 wt%, while the concentration of PS was adjusted to get films of similar thickness (~300 nm) (2) Deposit the solution at room temperature (23 °C) by spin-coating (3000 rpm, 30 s) on a DCG grating (RB) or on a FS substrate (RT). (3) Remove toluene by thermal annealing at 90 °C for 30 min in an oven

## Data Availability

The data supporting the findings of this manuscript are available from the corresponding authors upon reasonable request.

## Supplementary Materials

The following are available online at https://www.mdpi.com/article/10.3390/polym13213843/s1, Figure S1: Electric field intensity distribution of the fundamental transverse electric mode and refractive index for DFB lasers with different configurations. Figure S2: DFB intensity versus the number of pump pulses for the DFB lasers with different concentrations of TPD in PS in the active film for RB and RT configurations.
