# Supplementary material for: N,N′-Bis(3-methylphenyl)-N,N′-dyphenylbenzidine Based Distributed Feedback Lasers with Holographically Fabricated Polymeric Resonators
Source: Polymers (Basel). 2021 Nov 6;13(21):3843. doi: 10.3390/polym13213843 (PMC8587923; doi:10.3390/polym13213843)
Supplement: Supplementary file 1 [file polymers-13-03843-s001.zip › polymers-1421678-SI.pdf]

N,N´-bis(3-methylphenyl)-N,N´-dyphenylbenzidine based distributed feedback lasers with holographically fabricated polymeric resonators

Víctor Bonal [1], José A. Quintana [2], José M. Villalvilla [1], Pedro G. Boj [2], Rafael Muñoz-Mármol [1], Jose C. Mira-Martínez [1], and María A. Díaz-García [1,*]

[1] Departamento de Física Aplicada and Instituto Universitario de Materiales de Alicante, Universidad de Alicante, 03080 Alicante, Spain; victor.bonal@ua.es (V.B.); jmvs@ua.es (J.M.V.); rafa.marmol@ua.es (R.M.-M.); jcmm24@alu.ua.es (J.C.M.-M.)
[2] Departamento de Óptica, Farmacología y Anatomía and Instituto Universitario de Materiales de Alicante, Universidad de Alicante, 03080 Alicante, Spain; ja.quintana@ua.es (J.A.Q.); p.boj@ua.es (P.G.B.)
* Correspondence: maria.diaz@ua.es

Keywords: Polymer films; Organic distributed feedback laser; Laser threshold; Photostability

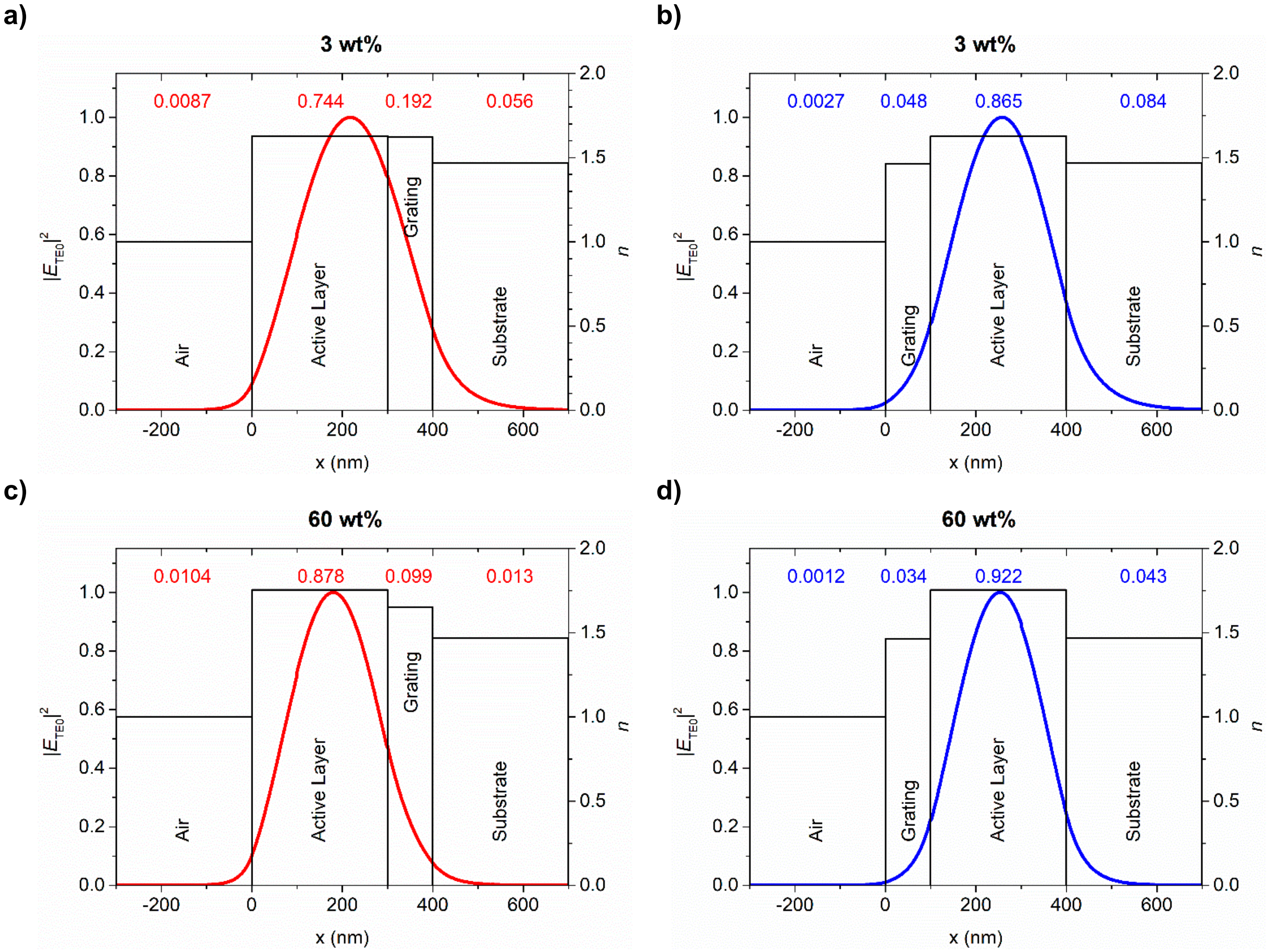

Figure S1. Electric field intensity distribution of the fundamental transverse electric (TE_0) mode (left axis) and refractive index (right axis) for DFB lasers with different configurations, RB for figures a) and c), RT for figures b) and d), and different TPD concentrations in the active film, 3 wt% for a) and b), 60 wt% for c) and d).

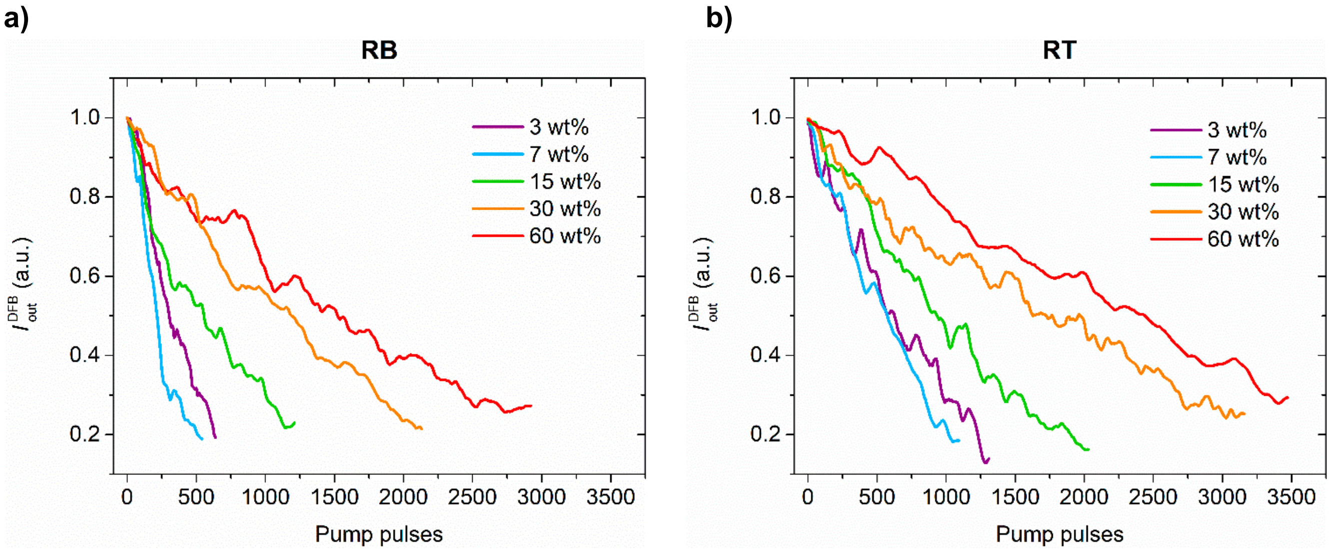

Figure S2. DFB intensity, I_{out}^{DFB}, versus the number of pump pulses for the DFB lasers with different concentrations of TPD in PS in the active film (from, 3 wt% to 60 wt%) for two different configurations: a) RB and b) RT. The operational lifetime ($\tau_{1/2}^{DFB}$) for each case is defined as the number of pump pulses at which I_{out}^{DFB} decays to half of its initial value.
